# Epidemiological profile, cardiopulmonary fitness and health-related quality of life of patients with heart failure: a longitudinal study

**DOI:** 10.1186/s12955-020-01634-3

**Published:** 2021-04-23

**Authors:** Paula Cristina Silva, Omar Pereira de Almeida Neto, Elmiro Santos Resende

**Affiliations:** 1 Health Sciences, Federal University of Uberlândia. CV, Rua Adamastor Leocádio, 624/ Bairro Pampulha, Uberlândia, Minas Gerais 38408-678 Brazil; 2 Health Care, Faculty of Medicine, Federal University of Uberlândia, Uberlândia, Minas Gerais Brazil; 3 Cardiology, Faculty of Medicine, Federal University of Uberlândia, Uberlândia, Minas Gerais Brazil

**Keywords:** Heart Failure, Quality of life, Physical limitation, Cardiorespiratory function

## Abstract

**Background:**

Heart failure (HF) is a severe and self-limiting syndrome. Its signs and symptoms are believed to predict poorer health-related quality of life scores, which are mainly influenced by deterioration in physical capacity. In the present study we try to analyze the influence of clinical and socioeconomic characteristics and physical capacity on the quality of life of people with HF diagnosis.

**Methods:**

A longitudinal study was conducted over 2 years with patients diagnosed with HF. To evaluate the patients the method of face-to-face visit and telephone monitoring was used. In the evaluations were applied: the Clinical and Socioeconomic Characterization Questionnaire, the Minnesota Living With Heart Failure Questionnaire (MLHFQ) for quality of life evaluation and the Veterans Specific Activity Questionnaire (VSAQ) for cardiopulmonary fitness analysis. Measures of central tendency, proportion, normality test, confidence intervals, comparison of data through paired Student t test and Wilcoxon or Mann Whitney test were performed and correlations were verified through Spearman coefficient.

**Results:**

The study included 108 patients, most of them female (50.90%) and mean age of 66.62 ± 11.33 years. The median time of HF diagnosis was 5 ± 6 years, being Chagas’ disease the main etiologic cause for the disease (57.40%). As for the clinical condition, functional classes II (44.40%) and III (48.10%) of the New York Heart Association (NYHA) were the most frequent. There was a low cardiopulmonary fitness, with loss of capacity to perform daily activities (3 ± 1 to 3 ± 3) over the time of clinical follow-up.

There was an increase in the MLHFQ instrument scores, from 50.98 ± 15.52 to 61.76 ± 19.95, over the analysis time. The analysis of correlations demonstrated that variables such as schooling, NYHA class, echocardiographic alterations and the drug profile have a significant relationship with the constructs of quality of life and physical fitness.

**Conclusion:**

Individuals in HF have significant impairment of cardiorespiratory capacity and tend to present worsening of QL along the evolution of the disease.

## Introduction

Cardiovascular diseases (CVD) rank first in the world’s leading causes of mortality. Among these diseases, heart failure (HF) represents an important cause of hospitalizations in elderly individuals, with increased morbidity and mortality rates and negative repercussions on socioeconomic status [1, 2].

This is a serious condition in which the heart becomes incapable of performing its blood ejection functions with reduction of cardiac output, or only performs them at the expense of increased filling pressures with increased pulmonary and venous systemic pressures. This situation generates damage to the aerobic capacity of the individual, manifested by dyspnea, fatigue, edema and, consequently, reduction of tolerance to physical effort, helping to establish the severity of the disease [3, 4].

The measurement of cardiopulmonary involvement imposed by HF has been recognized as an important clinical data to be considered in intervention plans aimed at reducing morbidity and mortality of these patients [4, 5]. Thus, the use of direct or indirect and low-cost clinical evaluation instruments are increasingly used [3, 5]. One of these evaluation tools is the stratification of HF in functional classes according to the level of tolerance to physical effort and the appearance of symptoms. This procedure was proposed by the New York Heart Association (NYHA) and the patients are identified, clinically, in four functional classes, being I those with structural heart disease, but still asymptomatic and IV those who have dyspnea symptoms even at rest, being classes II and III, intermediate [3]. Another method is the use of the Veterans Specific Activity Questionnaire (VSAQ), a questionnaire that proposes the clinical identification of aerobic and physical involvement of individuals with CVD [5].

The numerous changes in lifestyle and cardiopulmonary weakness appear as possible predictors of worse health-related quality of life (QVRS), a specific concept focused on the impact that the disease and/or treatment have on the subject [6, 7]. Therefore, it is believed that the analysis of QLRS associated to sociodemographic and clinical variables, is able to subsidize changes in care practices [3, 8].

In order to outline the physical impact of HF on the quality of life of sick individuals, this study aimed to analyze the clinical and sociodemographic profile, cardiopulmonary capacity and QVRS of HF patients in a school hospital throughout the evaluation time.

## Methods

This is an observational cohort study. Based on statistical records of outpatient cardiology care in a school hospital from 2010 to 2015, the total number of HF patients was 871 individuals.

The recruitment (T0) of individuals was performed in a medical consultation room provided by the university hospital, the patients were approached on the day of their pre-scheduled outpatient medical consultation, the objectives of the study were explained and the TCLE delivered to the volunteers for their signature.

**Fig. 1:**
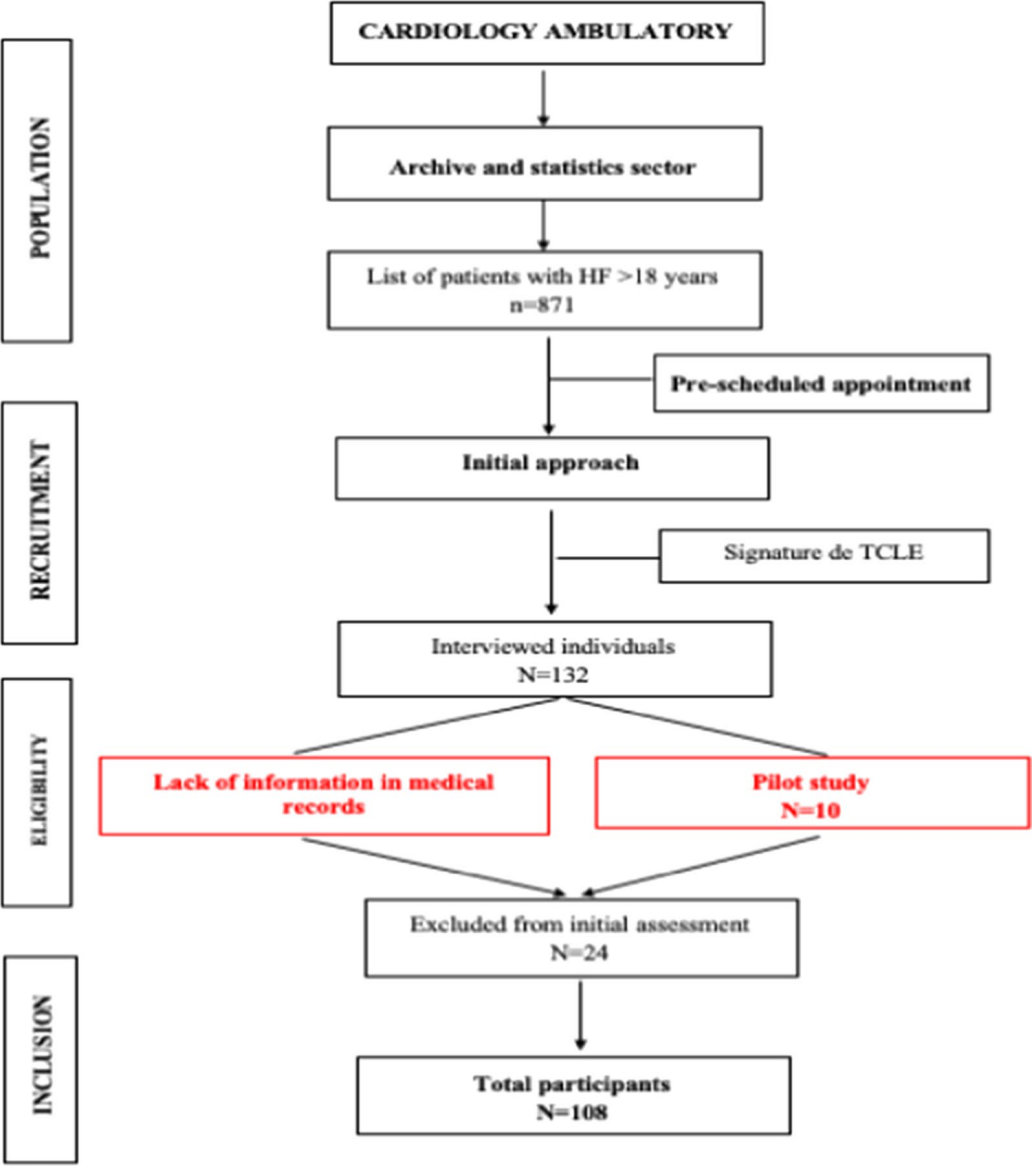
Flowchart of the data collection procedure

Eligible for the study were patients treated in the outpatient sector of a public university hospital, over 18 years of age and medical diagnosis confirmed in HF medical records. Individuals with recent history of hospital admissions (last 30 days) before the initial evaluation were excluded (Fig. 1).

Preliminarily, a pilot study was conducted with 10 patients to identify any difficulty that might exist in the study protocol [9].

### Collection process

**Fig. 2:**
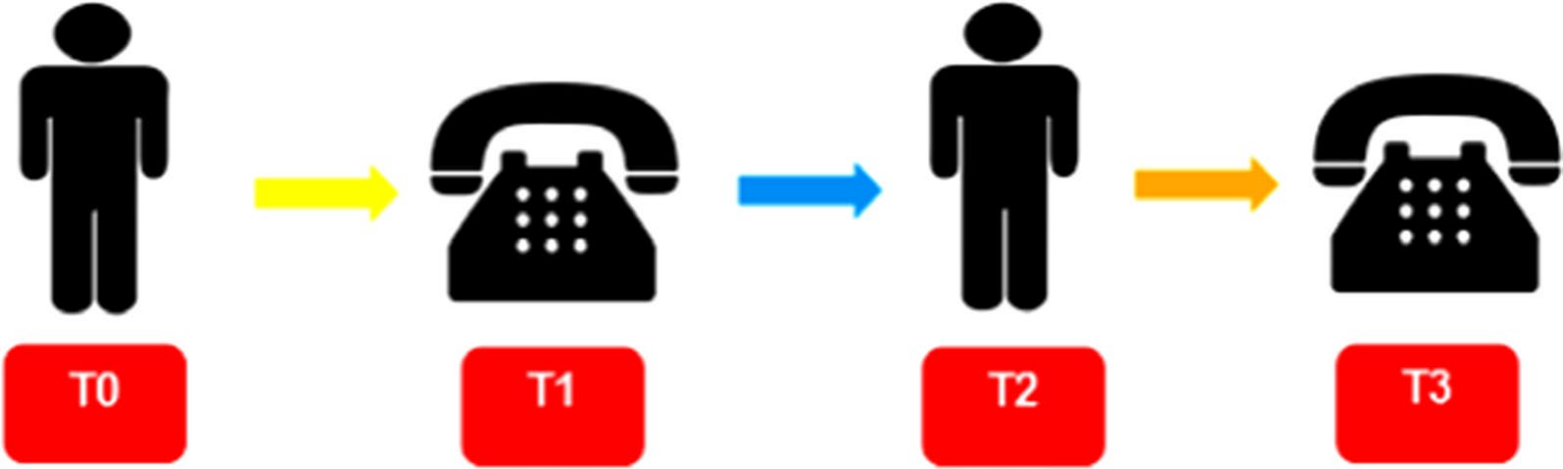
Flowchart of the semi-annual evaluation process, from December 2015 to December 2017. *T0 - Initial face-to-face evaluation; T1* - First telephone monitoring*; T2* - Second face-to-face evaluation; *T3 -* Second telephone monitoring

The data collection process occurred at four different moments with an average interval of 180 days between each evaluation, according to the models used by Mussi et al., 2013 [10] and Figueiredo et al., 2016 [11]. Figure 2 explains the process of data collection and longitudinal evaluation of participants.

The first approach (T0) occurred on the day of outpatient visits, the objectives of the study were explained and then, after signing the TCLE, a Clinical and Sociodemographic Characterization Questionnaire was applied [12]. The Brazilian version of “Minnesota Living With Heart Failure” (MLHFQ) was used in the evaluation of QVRS [13, 14]. Regarding the evaluation of cardiopulmonary fitness of heart patients, this was done indirectly through the VSAQ questionnaire, a short evaluative instrument composed of 13 levels of activities placed in progressive order of energy expenditure and measured in metabolic equivalents (METs) [5, 15].

The other evaluations (T1, T2 and T3), were carried out interspersed with telephone monitoring and face-to-face meetings, being employed in each phase all the evaluation instruments used in T0 [10, 11].

### Sample size

From the total population, the sample size was established through the coefficient of aprioristic determination (R2 = 0.13) [16], in a linear regression model with four predictors, having, as significance level, or type I error, α = 0.05 and type II error, β = 0.1, resulting in a statistical power of 90%. Using the Power Analisys and Sample Size (PASS) application, version 13, and introducing the above values, we obtained a minimum sample size of 108 subjects. The variable studied as main outcome was the QVRS score, in its physical domain (Fig. 1).

### Statistical analysis

Exploratory (descriptive) analyses of the data were carried out from the calculation of absolute simple frequencies and percentages for the categorical variables, the respective confidence intervals were also estimated for population proportion (p).

To represent the quantitative variables, the Shapiro-Wilk test (*P* > 0.05) first verified the normality, using the interval estimate of sample means and standard deviation, according to the presence of the normal distribution, as well as the confidence interval. In cases of absence of normality (asymmetric distributions) the median and the interquartile range (IQ) were estimated to represent the variability of the median and the confidence interval for the median.

The analyses were made using the Statistical Package for the Social Science (SPSS) software version 20.0, adopting a significance of 5% for all analyses.

In order to compare the data of the quantitative variables of the psychometric instruments, the total of individuals who completed the four moments of evaluation were considered, being calculated the increments for each of the participants, adopting the evaluated phase minus the initial phase. The increments were tested for zero mean, compared pairwise by paired Student’s t test or by Wilcoxon-Mann Whitney’s test, based on the presence or absence of normality, respectively [11]. In the analysis of the correlations between sociodemographic and clinical variables regarding aerobic fitness and QVRS, Spearman’s correlation was adopted and the hypothesis of nullity of correlations at the significance level (*p* < 0.05) with paired Student’s t test for the correlation coefficient was tested. The magnitudes of the correlations were classified as: weak (0 < r < 0.3), moderate (0.3 ≤ r < 0.5) and strong (*r* ≥ 0.5) [5, 11].

## Results

Of the 122 patients included in the study, fourteen had to be excluded from statistical analysis due to the absence of clinical information needed for the analysis in the medical chart. Therefore, at the end of T0, 108 patients were included in the sample, due to the longitudinal nature of the study, there were losses of participants, whose reasons were not attending the following evaluation appointments, not answering telephone monitoring calls in three alternate attempts and/or informed deaths, during the subsequent phases (T1, T2 and T3). For the comparison of the data of the quantitative variables of the psychometric instruments only the 68 (63.0%) were considered, participants who concluded the four evaluation periods.

### Socioeconomic characteristics

Based on the Clinical and Socio-economic Characterization Questionnaire, the average age of the patients was 66.62 ± 11.33 years, female (50.90%), married (54.60%), white (47.20%), with a degree of schooling of approximately 4 ± 6 years and average family income of R$ 1640.00 ± 1523.00.

### Clinical, echocardiographic and electrocardiographic characteristics

Table 1 presents the categorical clinical variables. A higher proportion of NYHA III functional class (*n* = 52; 48.10%), with chagasic etiology (*n* = 62; 57.40%) and among the most prevalent comorbidities hypertension (SAH), arrhythmia and coronary disease led the ranking.

**Table 1:** Clinical characteristics of patients (*n* = 108) with heart failure seen at the cardiology outpatient clinic in T0

VARIABLES	N	%	IC
**NYHA Functional Class**			
I	4	3,70	0,90-8,30
II	48	44,40	35,70-51,90
III	52	48,10	38,70-57,60
IV	4	3,70	0,90-8,30
**Etiology**			
Chagasic Cardiopathy	62	57,40	48,40-66,20
Ischemic heart disease	28	25,90	18,50-33,60
Hypertensive Cardiopathy	9	8,30	4,60-14,10
Valvar Cardiopathy	4	3,70	0,90-7,60
Idiopathic	5	4,60	0,70-8,30
**Comorbidities**			
Systemic Hypertension	91	84,30	78,50-90,30
Arrhythmia	43	39,80	30,60-47,20
Coronary Arterial Disease	32	29,60	23,10-41,00
Dyslipidemia	21	19,40	11,60-25,20
Angina	6	7,41	1,90-11,30
Diabetes mellitus	22	20,40	13,00-29,60
Atrial Fibrillation	18	16,70	9,30-24,30
Obesity	8	7,40	2,80-13,00
Stroke	6	5,60	0,90-10,20
**Drugs**			
Beta-blocker	93	86,10	78,70-92,60
Diuretic	81	75,00	64,60-82,40
IECA / BRA	71	65,70	57,00-75,40
Aldosterone antagonist	37	34,30	26,60-42,60
Antithrombotic	56	51,90	43,30-62,00
Statin	37	34,30	26,60-42,60
Digital	33	30,60	21,30-39,30
Antidepressant / Anxiolytic	19	17,60	10,00-25,20
Nitrate	9	8,30	4,20-15,00
**Cardiac Surgical Procedure**			
Pacemaker	80	74,10	64,80-83,30
Angioplasty	10	9,30	3,50-14,80
Revascularization	5	4,60	1,90-9,50
Valvoplasty	2	1,90	0,03-4,60
No surgical intervention	11	10,18	4,30-13,84
**Habits of Life**			
Smoking	13	12,00	6,00-18,50
Former smokers	28	25,90	16,40-34,50
Etilism	10	9,30	4,60-15,30
Ex-Etilists	22	20,40	13,00-26,90
Physical Activity	5	4,60	0,90-8,30
Cardiac Rehabilitation	1	0,90	0,03-2,80

*IC* Confidence interval

The time of diagnosis presented an average of 5 years.

Among the echo and electrocardiographic parameters, the majority of the participants had non sinus heart rhythm (*n* = 106; 98.10%), with marked presence of left branch block (BRE) (*n* = 98); 90.7%) and reduced left ventricle ejection fraction (LVEF) (40.90 ± 11.12%), associated with cardiovascular anatomical changes such as increased aortic diameter (34 ± 6 mm), left atrium (42.88 ± 5.35 mm) and left ventricle (60 ± 14 mm).

### Cardiopulmonary capacity of HF carriers

Table 2 represents the scores in METs of aerobic fitness achieved in the four different moments of evaluation (T0, T1, T2 and T3), by HF carriers.

**Table 2:** Cardiopulmonary fitness of patients with heart failure seen in the outpatient clinic

Times	Mín-Max	Median ± IQ	IC	***p***-value
**T**_**0**_ **VSAQ**	1–8	3 ± 1	3–3	< 0,001*
**T**_**1**_ **VSAQ**	1–11	3 ± 3	3–4	< 0,001*
**T**_**2**_ **VSAQ**	1–7	3 ± 2	3–3	< 0,001*
**T**_**3**_ **VSAQ**	1–7	3 ± 3	2–3	< 0,001*

*Min-Max* Minimum-maximum, *SD* Standard deviation; *p* < 0.05; *: indicate data that do not have a symmetric distribution; *IQ*: interquartile amplitude; *IC*: confidence interval. *T0* - Initial face-to-face evaluation; *T1* - First telephone monitoring; *T2* - Second face-to-face evaluation; *T3* - Second telephone monitoring.

### QVRS in IC

The QVRS scores reached in the four different moments of evaluation (T0, T1, T2 and T3) by the participants are shown in Table 3.

**Table 3:** Quality of life related to the health of patients with heart failure seen in the outpatient clinic

Times	Domains	Media ± DP	Median ± IQ	IC	***p-Value***
	Physical		25,50 ± 14	21,00-29,50	**0,009**
**T**_**0**_	Emotional	10,44 ± 5,65		9,05-11,83	0,250
	Unspecific	15,59 ± 4,44		14,50-16,68	0,153
	Total	50,98 ± 15,52		47,17-54,80	0,186
	Physical	24,59 ± 7,88		22,65-26,53	0,499
**T**_**1**_	Emotional	9,74 ± 5,00		8,51-10,97	0,439
	Unspecific	16,79 ± 5,00		15,56-18,02	0,181
	Total	51,12 ± 15,73		47,25-54,99	0,449
	Physical	25,80 ± 8,48		23,72-27,89	0,055
**T**_**2**_	Emotional		10,50 ± 9	7,88-13,00	**0,043**
	Unspecific		19 ± 6	18–20	**0,033**
	Total	54,79 ± 15,64		50,94-58,63	0,183
	Physical	28,64 ± 7,76		26,73-30,54	0,061
**T**_**3**_	Emotional		11 ± 10	10–14	**0,033**
	Unspecific	21,97 ± 5,09		20,72-23,22	0,731
	Total	61,76 ± 19,95		57,59-65,92	0,281

*SD* Standard deviation*; p <* 0.05; *: indicate data that do not have a symmetric distribution*; IQ* Interquartile amplitude, *IC* Confidence interval.

Table 4 explains the results obtained by each study participant in the MLHFQ and VSAQ questionnaires, over the follow-up time (T0, T1, T2 and T3), demonstrating through the calculation of increment and comparison the modification of the QVRS profile and aerobic fitness of HF patients with the evolution of the disease.

**Table 4:** Comparison of the evaluated constructs in patients with Cardiac Failure over the time of clinical follow-up (T0, T1, T2 and T3)

Variable	Average + EP	Median - IQ (n)	Statistics (***p***)
T1MINFIS	−0,29 ± 1,03	−1 - 10,5 (75)	*t* = − 0,28 (0,779)
T2MINFIS	0,78 ± 1,13	1–10,25 (72)	*t* = 0,69 (0,494)
T3MINFIS	4 ± 1,12	2–12,25 (68)	*t* = 3,56 (0,001)
T1MINEMO	−0,53 ± 0,72	0–7 (75)	*t* = − 0,73 (0,468)
T2MINEMO	−0,21 ± 0,81	0–9,25 (72)	*t* = − 0,26 (0,798)
T3MINEMO	0,91 ± 0,92	1–10 (68)	*t* = 0,99 (0,326)
T1MININES	0,99 ± 0,65	1–8 (75)	*t* = 1,52 (0,133)
T2MININES	3,06 ± 0,56	3–7 (72)	*t* = 5,42 (< 0,001)
T3MININES	6,56 ± 0,72	6–7,5 (68)	*t* = 9,07 (< 0,001)
T1MINTOTAL	0,17 ± 1,76	−0,5–19,5 (75)	*t* = 0,10 (0,923)
T2MINTOTAL	3,63 ± 1,88	4–24,5 (72)	*t* = 1,93 (0,058)
T3MINTOTAL	11,47 ± 2,29	10–24,75 (68)	*t* = 5,02 (< 0,001)
T1VSAQ	0,08 ± 0,17	0–2 (75)	*Z* = − 0,55 (0,584)
T2VSAQ	−0,17 ± 0,16	0–2 (72)	*Z* = 1,09 (0,276)
T3VSAQ	−0,25 ± 0,17	0–2 (68)	*Z* = − 1,59 (0,112)

*MINFIS* Physical domain*, MINEMO* Emotional domain*, MININES* Non-specific domain*, MINTOTAL* Total score*, EP* Standard error, *IQ* Interquartile range*, n* Number of participants*, t* Paired Student t test*, Z* Wilcoxon test, *p* Probability.

### Correlation between socioeconomic profile, therapeutic, QVRS and cardiorespiratory fitness

The correlation analyses showed a significant negative relationship of weak magnitude between schooling and the non-specific domain of MLHFQ. The categorical clinical variables of time of HF diagnosis, functional class and therapeutic profile correlated significantly with QLRS constructs and cardiopulmonary fitness (Table 5).

**Table 5:** Correlation of socioeconomic, clinical and drug domains and the QL and cardiorespiratory fitness constructs of participants at T0

VARIABLES	T0MINFIS	T0MINEMO	T0MININES	T0MINTOT	T0VSAQ
**Socioeconomic profile**					
Age	−0,069	0,023	−0,136	− 0,055	− 0,023
Schooling	− 0,038	− 0,176	**−** 0,240*	− 0,166	0,064
Civil Status	− 0,092	− 0,042	0,013	−0,089	0,015
Indiv. Income (R$)	−0,134	−0,05	− 0,096	−0,133	0,140
Family Income (R$)	−0,037	−0,186	0,046	−0,071	0,147
**Clinical Profile**					
Diagnostic Tempo	− 0,028	− 0,048	− 0,215*	−0,092	0,067
NYHA	0,295^**┼**^	−0,002	0,222*	0,245*	−0,209*
LVEF	0,035	0,115	−0,139	0,011	−0,023
**Drug Profile**					
Beta-blockers	0,049	0,034	0,106	0,067	0,037
Antithrombotics	0,133	0,156	0,124	0,160	0,209*
Diuretics	0,113	0,024	0,169	0,098	−0,068
IECA/BRA	0,004	−0,034	0,114	0,010	0,092
Ant. Aldosterone	−0,06	−0,09	0,110	0,070	0,060
Digitals	−0,021	0,036	0,021	0,041	−0,080
Nitrate	0,023	0,034	0,036	0,056	−0,023
Statins	−0,078	0,032	0,047	−0,021	0,047
Antidep./Ansiol.	0,095	0,151	0,104	0,149	−0,213*

*MINFIS* Physical domain, *MINEMO* Emotional domain*, MININES* Non-specific domain, *MINTOTAL* Total scan, *VSAQ* Veterans Specific Activity Questionnaire. *: *p* < 0.05; ┼: *p* < 0.01; *t* test of Student *Paired.*

## Discussion

### Socioeconomic characteristics

In the analysis of a cohort of patients with HF like the one in the present study, the finding of a larger contingent of elderly is not uncommon and is corroborated by numerous studies [7, 10, 12, 17, 18] and justified by the increase in life expectancy, advances in the field of health with the incorporation of integral and multiprofessional action therapies and the decline in birth rates [17, 18]. Also the greater presence of female patients, although women are less affected by heart disease [7, 13, 15, 18], is justified, according to Pilger, 2011 [19] and Albuquerque et al., 2016 [20], as a reflection of their greater engagement in preventive health strategies, better adherence to the established treatment, facts that generate fewer events of decompensation of HF when compared to men [13].

The low schooling and economic condition of the patients in this study may directly interfere in the access to health services, self-care capacity and adherence to treatment, contributing, besides HF, to the decline in quality of life [7, 11, 13, 21].

### Clinical characteristics, echocardiographic and electrocardiographic variables

The NYHA II and III classes of HF presented higher prevalence in the present study, a fact that can be linked to the longer time for diagnosis and to aspects of the established treatment [7, 12, 13]. The diagnosis and the beginning of treatment annul the disease, when late, print a greater functional impairment, implying worsening of HF and presentation of advanced classes of NYHA, which makes the control of the disease more difficult and costly.

It is estimated that the survival rate after the diagnosis of HF is around 5 years, thus indicating that this disease has a worse prognosis than many types of cancers, especially if HF is allied to reduced LVEF [2, 22].

Regarding the clinical characteristics, the anatomical changes of the heart result from the remodeling process of the cardiovascular apparatus present in heart patients and this compromise may imply in the reduction of cardiac output [13, 23]. It is believed that this condition is also associated with the main etiology of HF found in this study, the chagasic heart disease, which still presents high prevalence in our environment and evolves with systolic dysfunction resulting from myocardial fibrosis [24, 25], bradyarrhythmias and electrical conduction disorders, often configuring the need for implantation of cardio-stimulators and electrical cardioverters [21]. This aspect with regional characteristics [25, 26] has caused our cohort to present a large number of patients with chagasic and pacemaker, differently from what has been reported in other studies in which ischemic heart disease is the main cause of HF [7, 16, 24].

Among the comorbidities associated with HCI, HAS is the most common in our study. Hypertension increases the systolic overload imposed on the left ventricle and, consequently, induces hypertrophy with progressive deterioration of contractile function [13, 27, 28].

For the treatment of HF, the use of beta-blockers, diuretics, IECA/BRA found in this study corresponds to that recommended by the Brazilian Guidelines of Chronic Heart Failure. However, the percentage of IECA/BRA prescriptions and aldosterone antagonists are lower than what is considered ideal in the treatment of the disease. This non adherence to the guidelines has been the object of studies and may be due to the lack of medication available in the public system, a very important factor because we are treating patients with low purchasing power. Another justification is the frequent presence of chronic renal disease in heart patients. Many professionals have an understanding that these drugs should not be used in these conditions and, therefore, their use is restricted in clinical practice [23, 24].

### Cardiorespiratory fitness in patients with HF

In the present study, HF carriers had an estimated performance in METs lower than those found in the literature [29–31], indicating a greater severity of the disease (VO2relative < 18 ml/kg/min). It is known that values lower than 4 METs are related to reduced survival and constitute one of the indications for heart transplantation [19, 32].

When comparing the scores of the VSAQ throughout the follow-up of patients, the time of evolution of HF and poor adherence to more healthy living habits are predictors of the decline in functional capacity of the individual, since the low adherence to physical activity and cardiac rehabilitation programs contribute to mitigate the deterioration of individual autonomy and muscle atrophy and this inactivity leads to aerobic damage, especially in elderly subjects [23].

### QVRS of patients with HF

The QVRS estimated according to signs, symptoms and therapy established for the disease, revealed worsening in the physical domain throughout the follow-up of patients, and the scores obtained were higher than those found in other studies [7, 16, 33, 34]. Hypothetically, this finding may be related to the fact that the most frequent functional classes of HF in this study were II and III, with symptoms ranging from edema and fatigue, to more intense dyspnea to small efforts.

It is known that the signs of a certain illness directly influence the emotional construct [29, 30], resulting in introspection, reduction of leisure activities, early retirement, loss of autonomy and independence [12, 29]. The greater severity of HF in our patients is implicated in the evaluation performed.

Factors such as side effects resulting from the therapy adopted in the treatment and the necessary changes in lifestyle may justify the significant growth of the score in the non-specific domain from T0 to T3 [21, 23]. Although this domain is little discussed in the literature, it is known that the economic situation of the individual tends to change with the progression of the disease, implying difficulties in adherence to treatment, access to health service and, consequently, decline in QLRS [21].

The increased total score configures a significant worsening of the quality of life, inferring the impact of the degenerative characteristics of HF in the social and financial context, adherence to treatment and changes in living habits that arise with the evolution of heart disease [21, 22]. When compared to the follow-up time, this significant increase suggests that the attention to HF is still a public health challenge because, as it is known, there is a direct relationship between worsening quality of life and increased mortality rates in HF [7, 23].

### Correlation between socioeconomic profile, therapeutic, QVRS and cardiorespiratory fitness

The negative and significant correlation between schooling and worsening in the non-specific field of MLHFQ confirms the importance of educational actions in health in the treatment of HF. The level of education favors therapeutic engagement and the adoption of healthy living habits, so that schooling low distances the individual from the ideal treatment for HF and culminates in the worst evolution of the disease [21, 23].

The time of diagnosis of HF, a topic little addressed in clinical discussions, is important in the prognosis of the disease. With the identification of HF, the individual is forced to move away from the labor market, have additional spending on medication and promote changes in living habits, which immediately generates fear for the unknown and therefore justifies its significant and negative correspondence with the general dimension of MLHFQ [6, 7].

The NYHA functional classification presents a positive and significant correlation with the physical, non-specific and total dimensions of the QL questionnaire. Advanced NYHA classes correspond to a greater physical impairment that interferes with the development of daily life activities due to the presence of disease symptoms. Thus, the scores in MLHFQ tend to be higher [10].

In relation to VSAQ, the functional capacity of the individual demonstrates a significant and inverse correlation with the values obtained. The most advanced class of NYHA, as already mentioned, is accompanied by exacerbated signs and symptoms of HF reflecting exponential reduction of aerobic capacity of the individual [5, 15, 23].

In view of the pharmacological profile, individuals using antithrombotics and anxiolytics have a correlation with cardiopulmonary fitness. The antithrombotics, indicated due to the increased risk of ischemic events, predispose to better physical capacity and significant reduction of the functional class, reducing rates of infarctions and strokes, while the antidepressants and anxiolytics are known to generate lethargic conditions, affecting the physical disposition and causing aerobic damage over the time of evolution of HF [24, 25].

## Limitations

The follow-up of all clinical characteristics during the four evaluation periods could not be performed. It is believed that such data would be valuable for broader and more solid clinical and scientific judgment.

Another limitation that should be considered is the rate of patient loss throughout the study by reducing the sample number at each evaluation time.

Despite these limitations, this is the first Brazilian study using an indirect instrument of relative aerobic capacity measurement in HF patients and it was possible to validate it as a potential tool for clinical practice, subsidizing the decision making of the multiprofessional team in the management of the disease in order to preserve the autonomy and independence of the individual.

## Conclusion

HF, characterized as a chronic-degenerative disease of high prevalence in our environment, presents, in its evolution, progressive morphofunctional changes of the cardiovascular system. These epidemiological aspects, added to the socio-demographic profile of low schooling and reduced purchasing power of the patients, configure a great challenge in the management of resources and overload the health actions.

Since the beginning of the study, the participants were already severely ill, in advanced functional classes of HF and with low aerobic capacity, which may have compromised the physical, social and psychoemotional scores, resulting in a worse evaluation of the quality of life. The progressive deterioration of QVRS can be gauged throughout the study.

It is also known that negative changes in living habits and low quality of life, imply in a worse prognosis of the disease and should guide new methods of preventive and therapeutic approach of HF.

## Data Availability

All data sets analyzed during the current study that were not presented in the texts are available to the correspondent upon request.

## Abbreviations

BRA: Angiotensin receptor blocker
BRE: Left branch lock
CVD: Cardiovascular diseases
HAS: Systemic hypertension
HF: Heart Failure
QVRS: Quality of life related to health
IECA: Angiotensin conversion enzyme inhibitors
LVEF: Left ventricle ejection fraction
METs: Metabolic Equivalent
MLHFQ: Minnesota Living With Heart Failure Questionnaire
NYHA: New York Heart Association
TCLE: Informed consent form
VSAQ: Veterans Specific Activity Questionnaire
